# Technoeconomic Analysis of a Novel Microwave Process to Produce Ethylene from Methane

**DOI:** 10.1021/acsomega.5c11191

**Published:** 2026-01-26

**Authors:** Md Mizanur Rahman, Md Emdadul Haque, Snehitha Reddy Baddam, Jianli Hu, Srinivas Palanki

**Affiliations:** Department of Chemical and Biomedical Engineering, 5631 West Virginia University, Morgantown, West Virginia 26506, United States

## Abstract

Ethylene is a critical feedstock in industrial processes, serving as a raw material in the petrochemical industry to produce plastics and commodity chemicals. Conventional ethylene production routes are energy-intensive and contribute substantially to the carbon footprint of chemical manufacturing. In this study, an industrial-scale novel microwave process was simulated using ASPEN Plus to assess its economic viability. Technoeconomic analysis confirmed the economic competitiveness of the novel microwave process, with a levelized cost of ethylene of USD 0.51/kg compared to USD 0.56/kg for the conventional base case. Key economic drivers of the process were identified, and sensitivity analyses were conducted to evaluate their impact on project economics. Furthermore, 87.7% of the total utility consumption in the novel microwave process is electricity, highlighting its potential to contribute to the electrification of the chemical industry. The findings of this study confirm the economic feasibility of both industrial-scale microwave reactors and modular plant configurations for the production of ethylene from methane, offering a promising alternative to conventional processes.

## Graphical abstract

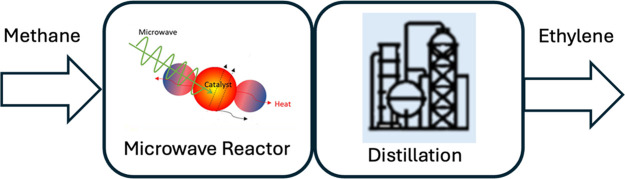

## 1 Introduction

Ethylene is a critical feedstock extensively utilized across a wide range of industrial applications and serves as a foundational building block for the synthesis of polymers such as polyethylene and polyvinyl chloride (PVC), as well as commodity chemicals such as ethanol, ethylene oxide, and ethylene glycol.^1,2^ Global ethylene production is experiencing a compound annual growth rate of 6.2%,^3^ reaching 228.53 million tons in 2023.^4^ Conventionally, ethylene is synthesized through the steam cracking of naphtha, a process that entails the thermal decomposition or pyrolysis of naphtha at elevated temperatures.^2,5–8^ The advent and widespread adoption of horizontal drilling and hydraulic fracturing technologies for shale gas extraction have resulted in an abundant supply of ethane, thereby shifting the industry’s feedstock preference from naphtha to ethane for ethylene production via noncatalytic cracking.^9,10^ Alternatively, the methane feedstock from shale gas is being utilized to first make methanol and then converted to ethylene via methanol-to-olefins (MTO) pathways.^11^ These traditional production routes are characterized by high energy consumption and significant carbon emissions,^2,12–14^ largely due to the inherently endothermic nature of the reactions, which typically require temperatures exceeding 800 °C.^5,6,10,14^

To address these challenges, process intensification strategies are being explored in conjunction with the electrification of process heating technologies to improve energy efficiency and reduce the environmental footprint of ethylene production, thereby supporting the transition toward more sustainable chemical manufacturing practices.^8,15,16^ In this context, the direct conversion of shale gas to ethylene through oxidative coupling of methane (OCM) has emerged as a promising alternative.^11^ For instance, Delikonstantis et al.^17^ demonstrated that the electrification of reactors can enable efficient ethylene production via methane (CH_4_) cracking. Similarly, Julian et al.^15^ explored process intensification using microwave-assisted catalytic reactors and demonstrated their potential as a viable alternative for methane conversion. Electrification of ethylene production processes offers significant advantages in terms of both energy efficiency and reduction of carbon emissions.^3^ In particular, the application of microwave (MW) reactor technology is aimed at lowering energy consumption and minimizing environmental impact by leveraging novel, alternative processing routes.^18^ Under microwave irradiation, methane can be directly converted into ethylene at comparatively lower temperatures.^19^ Although significant advancements have been made at the laboratory scale in developing microwave-assisted technologies as potential alternatives to conventional ethylene production routes, their large-scale industrial implementation and the corresponding economic feasibility of ethylene synthesis from methane using microwave reactors have been inadequately explored. This knowledge gap motivates comprehensive technoeconomic analysis of a microwave-assisted process for the large-scale production of ethylene from methane.

The objective of this study is to quantitatively evaluate the economic performance of this emerging technology, thereby providing insights into its potential viability as a sustainable alternative to conventional ethylene production pathways. Prior research has largely focused on reaction feasibility under microwave conditions without assessing whether such a process can be economically viable at scale. The proposed process is novel because it enables ethylene production through a nonequilibrium, continuous pathway powered by microwave heating, rather than conventional thermal methods. The reactor configuration, microwave frequency, and catalyst system differ significantly from prior studies,^15,17^ and move beyond typical lab-scale demonstrations by introducing a three-reactor series configuration and evaluating its feasibility for industrial-scale methane-to-ethylene conversion, a level of integrated process simulation not previously reported. To the best of our knowledge, this is the first study to evaluate the technoeconomic performance, engineering design requirements, energy-integration strategies, operating needs, and cost competitiveness of scaling up MW-assisted methane conversion for ethylene production. By filling this gap, the present work links fundamental MW-driven chemistry to the practical considerations needed for industrial process design and implementation.

First, a base case simulation of a conventional plant that produces ethylene from methane via the MTO process was developed in the ASPEN Plus environment. This simulation was based on Natgasoline’s production process in Beaumont, TX.^20^ Then, laboratory-scale experimental data from a MW-assisted catalytic reactor that produces ethylene from methane were used to develop a process flowsheet that utilizes the same feed as Natgasoline’s conventional process, along with a separation scheme to produce polymer-grade ethylene (99.9%). This novel process was simulated in the ASPEN Plus environment, and a technoeconomic analysis (TEA) was conducted and compared with the conventional natural gas to ethylene industrial process.

## 2 Methodological Framework

A steady state simulation model was developed of the conventional process and microwave-assisted novel process by ASPEN Plus v14.0. A heat exchanger network (HEN) was designed applying ASPEN Energy Analyzer (AEA) v14.0 to maximize heat recovery from hot and cold utilities to optimize energy consumption.^20–22^ Finally, ASPEN Process Economic Analyzer (APEA) v14 was used for TEA based on simulation specifications.^20,22–24^ Initial equipment and installation costs, as well as contingencies, were calculated to evaluate capital expenditure (CAPEX), and raw material, utilities, maintenance, and other costs to estimate operational expenditure (OPEX). The net present value (NPV) was calculated at a discounted rate to evaluate the project's profitability.

A comprehensive model of the base case was developed for syngas production from methane, methanol synthesis from syngas, followed by ethylene production from methanol, and finally a train of distillation columns for purification of the desired products. For the conventional methanol-based route, the nonrandom two-liquid (NRTL) model was chosen because it provides accurate predictions for highly nonideal, polar, and associating liquid mixtures, such as methanol–water and methanol–hydrocarbon systems commonly encountered in the MTO process. NRTL is widely utilized for alcohol-containing mixtures and reliably models liquid-phase nonideality during methanol synthesis, purification, and subsequent olefin production.^25^ For this base case, a methane feed rate of 67.781 t/h was used in the simulation, where ethylene is the main product and propylene is the byproduct. Process specifications, operating conditions, and reaction kinetics were gathered from Natgasoline’s process plant data.^20,26^ The same methodological approach was utilized for developing a steady-state simulation of the novel microwave process to produce ethylene from methane^20^ using the same feed rate and feed conditions of methane as the conventional process. For the MW-assisted methane conversion process, the Soave–Redlich–Kwong (SRK) equation of state was selected because this process primarily involves light hydrocarbons (CH_4_, C_2_H_4_, C_2_H_6_), H_2_, CO, CO_2_, and gas-phase reaction conditions. SRK is well established for predicting vapor-phase thermodynamic properties of nonpolar and mildly polar gases and provides robust performance for hydrocarbon reforming, cracking, and high-temperature reactors.^25^ Economic parameters of both the conventional and the novel microwave process were compared. Details of the process, economic analysis, and factors affecting the economy are discussed in successive sections.

## 3 Steady-State Simulation Model Development

### 3.1 Base Case Simulation Process of Ethylene Production from Natural Gas

Figure 1 presents the process flow diagram (PFD) for ethylene production from shale gas, based on the conventional Natgasoline process reported by Haque et al.^20^ The simulation employs the NRTL thermodynamic property model^27^ and is briefly described here. The process begins with the conversion of a methane feed (67.781 t/h) into synthesis gas, followed by its transformation into methanol, and subsequently into olefins.^20^ Reactor R-01 facilitates hydrogenation at 380 °C and 50 bar, while R-02 serves as the desulfurization reactor operating at 375 °C and 48 bar. The steam reforming section comprises three reactors, R-03, R-04, and R-05, responsible for converting the feed into CH_4_, H_2_, and CO_2_. The operating conditions are 449 °C and 48 bar for the prereformer (R-03), 730 °C and 36 bar for the steam reformer (R-04), and 975 °C and 35 bar for the secondary reformer (R-05).^20^ Following gas separation and compression, the process stream is directed to the methanol synthesis unit, which includes two parallel reactors (R-06A and R-06B) operating at 254 °C and 76 bar to produce methanol via the dehydrogenation of CO and CO_2_. The final conversion occurs in reactor R-07 at 220 °C and 76 bar.^20^ Methanol purification to 99.99% purity is achieved through three distillation columns (C-01, C-02, and C-03). The purified methanol is subsequently converted to olefins in reactor R-08 at 540 °C and 1.22 bar. The resulting product stream is fractionated through a series of distillation columns, with C-04 separating ethylene and propylene, C-05 isolating the methane-rich stream, and C-06 yielding 29.135 t/h of 99.9% pure ethylene. Additionally, C-07 produces approximately 18.914 t/h of propylene as a byproduct in this conventional process.

**Figure 1:**
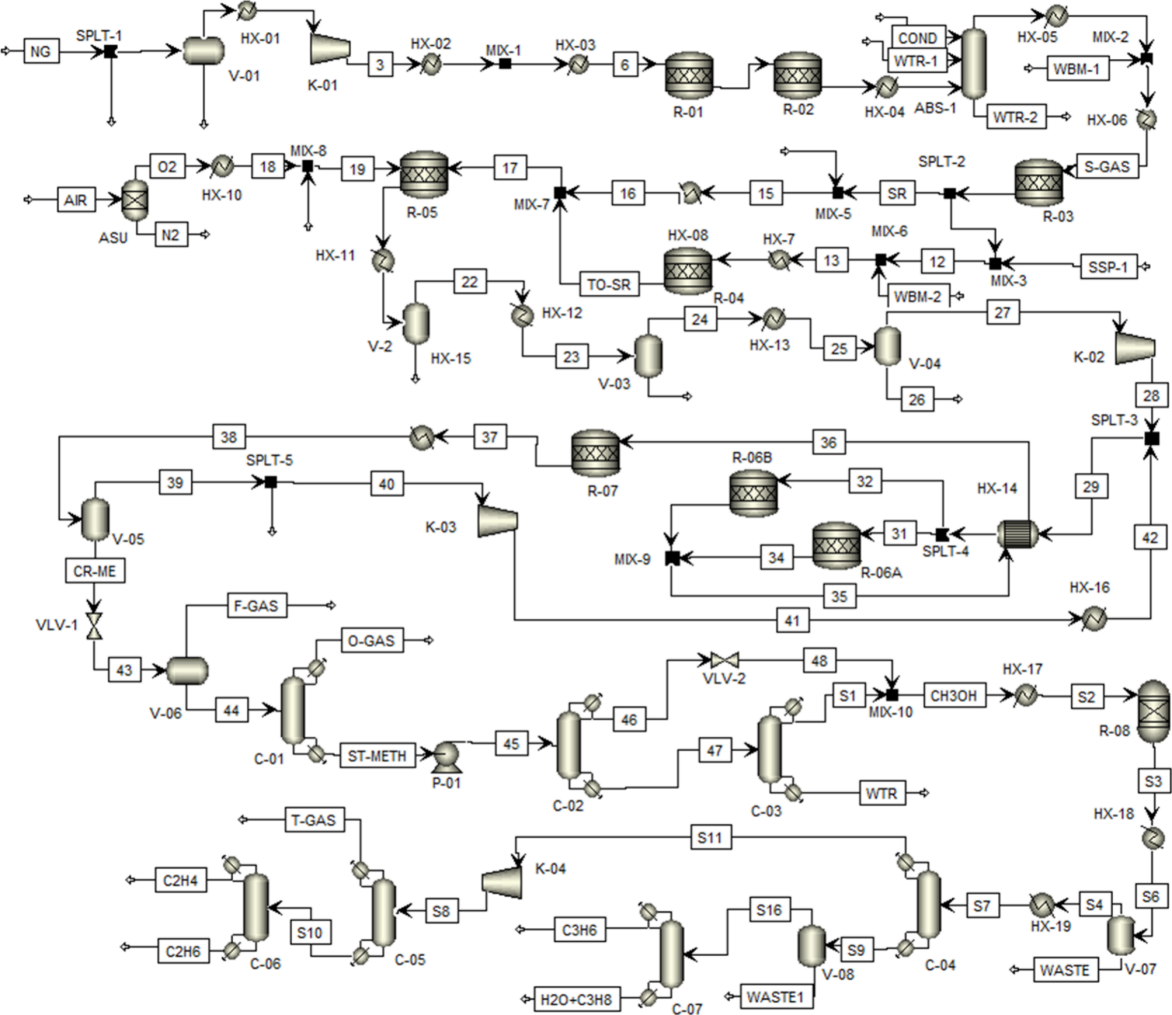
Process flow diagram of the conventional process for methane to ethylene.

### 3.2 Microwave Enhanced Catalytic Conversion of Methane to Ethylene

To scale laboratory reactor data to industrial conditions, several necessary assumptions were made while developing the ASPEN Plus simulation model, which may affect the reliability and generalizability of the technoeconomic results. The effectiveness factor, product composition, and microwave power efficiency observed at the lab scale were assumed to remain unchanged at the industrial scale. Likewise, catalyst activity, selectivity, and nonequilibrium microwave heating effects were treated as directly scalable, and the simulations also assumed uniform heating and ideal mixing. These assumptions are valid because we assumed that the microwave reactor module consisted of several units of a 200 kW microwave module, which is currently available commercially. This numbering-up of smaller microwave modules that are currently available, rather than the use of a single scaled-up microwave reactor, ensures that the laboratory-scale reactor conditions are not very different from the industrial-scale system, and hence the overall conversion observed in the laboratory scale is also valid at the larger scale. Table 1 presents the product distribution obtained from a laboratory-scale microwave-assisted catalytic reactor used for the conversion of methane to ethylene.^28^ Based on these results, the composition found from the conversion data and product distributions was directly incorporated into Aspen Plus using a yield reactor, ensuring consistency with observed laboratory performance. Experimental results show a methane conversion of 14.4% at a methane feed rate of 0.080357 mol/h. The w8 hly space velocity (WHSV) of methane in the system is 60 standard cubic centimeters per minute (SCCM) per gram of catalyst.^19^ Catalyst deactivation, coke formation, and reactor fouling can lower methane conversion and product selectivity, increasing operating costs due to shutdowns for cleaning or regeneration. These challenges can be mitigated using standby reactors or inline catalyst regeneration to maintain continuous operation, which is standard practice in industrial packed-bed reactors. The reactor generates a substantial quantity of ethane and acetylene as byproducts; therefore, the resulting product stream is directed to a secondary microwave reactor, where ethane is further converted to ethylene. In this reactor, the ethane conversion is 91.6%, with an ethylene yield of 55% at a temperature of 650 °C and a pressure of 1 atm.^29^ Subsequently, the outlet stream from the second reactor is introduced into another reactor designed for the selective hydrogenation of acetylene to ethylene.^30^ This reactor achieves complete (100%) conversion of acetylene to ethylene at 280 °C and 1 atm pressure.^31^ The experimental data obtained from these laboratory-scale systems form the foundation for developing a steady-state industrial-scale simulation using ASPEN Plus v14.0. It is important to note that industrial-scale implementation of MW-assisted reactors requires new policy adaptations, including guidelines for MW frequency exposure, operator and public safety, high-power electricity hazards, and safety measures, along with careful consideration of heating/cooling systems, high-voltage distribution, and safety interlocks to manage the added complexity compared with conventional fired systems.^32,33^ In the scaled-up microwave-assisted process, the methane feed rate is 67.781 t/h, consistent with that used in the conventional process. However, the product distribution obtained in the series of three microwave reactors differs significantly from that of the conventional process, necessitating a reconfiguration of the downstream separation system. Specifically, given the low conversion of methane, it is necessary to recycle the unreacted methane back to the microwave reactor, and the microwave-assisted process yields 44.076 t/h of ethylene and 7.35 t/h of pyrolysis gasoline (pygas). These factors have substantial implications for the overall process design and economic performance.

**Table 1:** Product Distribution from Microwave Reactor 1

component	mass fraction
CH_4_	0.8531
C_2_H_4_	0.0783
C_2_H_2_	0.0060
C_2_H_6_	0.0277
CO	0.0000
CO_2_	0.0006
C_6_H_6_	0.0025
C_7_H_8_	0.0013
H_2_	0.0212
coke	0.0093
total	1.0000

Figure 2 shows the process flow diagram of the novel microwave-assisted process to produce ethylene from methane. The SRK thermodynamic property model is used for this simulation.

**Figure 2:**
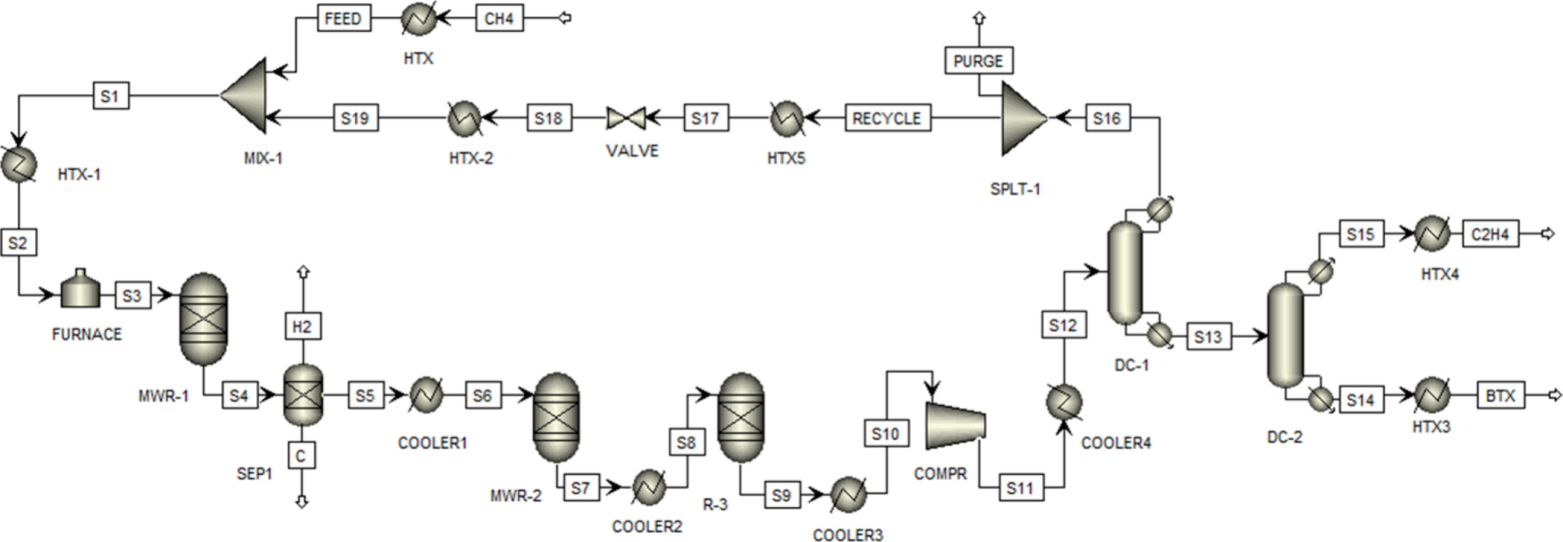
Process flow diagram of the MW assisted process for methane to ethylene.

The reaction conditions for the MW-assisted reactor are maintained at 700 °C and 1 atm in the presence of a MN–CeO_2_ catalyst. Prior to entering the reactor, the feed is preheated to 335 °C and subsequently heated in a furnace to the target reaction temperature of 700 °C. During the reaction, hydrogen and coke are formed as byproducts and subsequently separated. The gaseous effluent is then directed to a secondary dehydrogenation microwave reactor, where ethane is converted to ethylene. Under reaction conditions of 650 °C and 1 atm, the microwave reactor achieves an ethane conversion of 91.6% and an ethylene yield of 55%.^29^ For optimal methane conversion, the intermediate acetylene formed is further hydrogenated to ethylene at an optimal temperature.^30^ Consequently, the process stream is directed to a third reactor for the selective hydrogenation of acetylene to ethylene, achieving 100% conversion at 280 °C and 1 atm.^31^ The outlet temperature of the first reactor decreases from 700 to 650 °C, while the outlet stream from the second reactor cools from 650 to 280 °C. Two heat exchangers are integrated into the system to generate low-pressure steam for preheating the feed stream to the first microwave reactor, as illustrated in Figure 2. The recycle stream is heated to 278 °C before blending with the feed stream using HEN, developed and optimized via ASPEN Advanced Energy Analysis (AEA). The combined stream is compressed to 8 bar using two multistage 29280 kW electric-drive compressors to handle the low methane conversion (14.4%) and the resulting high recycle stream, which returns unreacted methane to the feed of the first MW reactor. This compressor size falls within the typical capacity of 41,200 kW.^34^ Then the mixture was cooled to −100 °C using an appropriate coolant before being fed to the first cryogenic demethanizer distillation column (C-1), which comprises 21 stages and separates CH_4_ and H_2_ to facilitate ethylene purification in the subsequent column. The top product from C-1, exiting at −129 °C, is sent to a splitter, where a portion is purged while the remainder is recycled to enhance the overall methane-to-ethylene conversion efficiency. The purge stream prevents the accumulation of inert components and ensures process efficiency, product purity, and operational stability, mitigating issues such as catalyst deactivation and reduced reactor performance. The bottom stream of column C-1, exiting at −57 °C, is directed to the second cryogenic distillation column (C-2) for ethylene separation, operating at 8 bar. The 57-stage column (C-2) yields polymer-grade ethylene (99.9% purity) as the top product at a flow rate of 44.076 t/h, while pygas is obtained as the bottom product at 7.3 t/h. The process simulation integrates a HEN to recover heat from various streams wherever applicable, based on AEA. As summarized in Table 2, the HEN configuration achieves a 42.48% reduction in hot utility consumption and an 89.08% reduction in cold utility demand, underscoring the significant energy efficiency improvements of the proposed MW-assisted process.

**Table 2:** HEN Performance

utility requirements (MMBtu/h)	base case	heat integrated	% reduction
hot utility	1119.21	643.79	42.48
cold utility	1261.03	137.73	89.08

**Figure 3:**
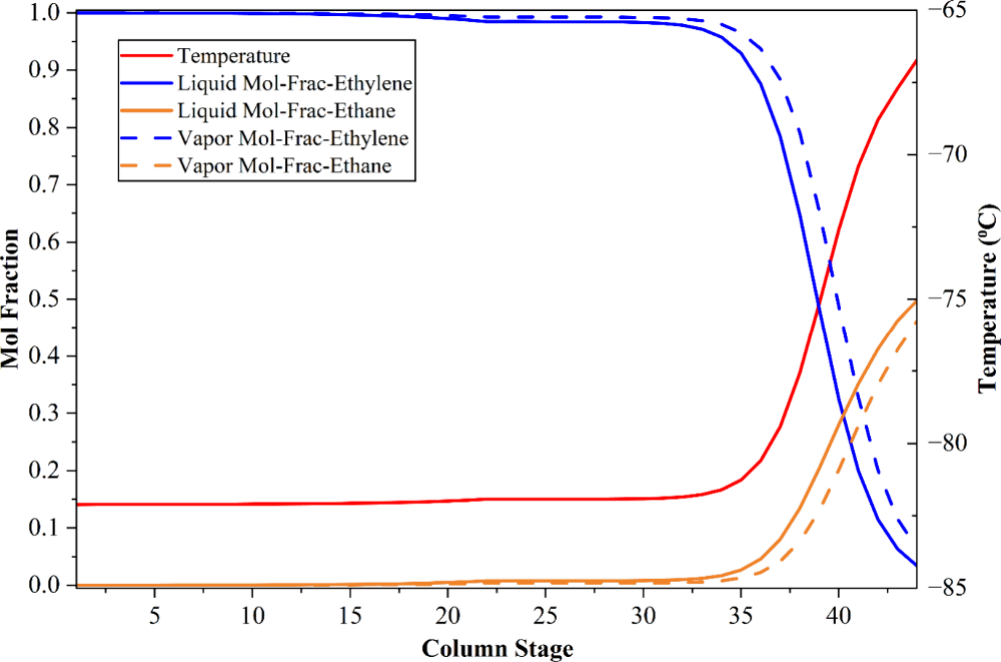
Temperature and composition profiles in the ethylene separator column of the conventional process.

## 4 Simulation Outcomes of the Separation Systems

### 4.1 Conventional Process

The distillation section in the conventional process consists of columns C-01, C-02, and C-03 for methanol purification, C-04 as a C_2_ splitter, C-05 serving as the demethanizer, C-06 for ethylene purification, and C-07 for propylene purification. The column design specifications for the simulated conventional process are summarized in Table 3. The demethanizer column (C-05) operates with a feed introduced at stage 5 of a seven-stage column and a reflux ratio of 8.6. For ethylene separation, the C-06 column features a feed stage at 22, a total of 44 stages, and a reflux ratio of 1.39, yielding polymer-grade ethylene at a flow rate of 29.135 t/h. In contrast, the propylene separation column (C-07) is designed with a feed stage at 20, a total of 36 stages, and a reflux ratio of 5.21, producing 18.914 t/h of propylene. The design parameters of these columns were optimized by adjusting the reflux ratio and the distillate-to-feed ratio to achieve the targeted ethylene and propylene purities.^20^ The simulation results indicate an ethylene purity of 99.9% and a propylene purity of 99.6%, confirming the effectiveness of the optimized separation design. Figure 3 illustrates the temperature-dependent vapor–liquid mole fraction profiles of ethylene and ethane within the ethylene separation column (C-06) of the simulated conventional process, whereas Figure 4 presents the temperature and composition profiles of propylene and butylene within the propylene separation column (C-07).

**Table 3:** Columns Specifications of the Simulated Conventional Process

	C-01	C-02	C-03	C-04	C-05	C-06	C-07
purpose	distillation						
no. of stages	12	47	30	8	7	44	36
feed stage	8	39	21	5	5	22	20
pressure (bar)	2.00	8.12	8.14	8.12	3.45	3.10	1.17
reflux ratio	1.82	1.27	1.02	2.28	8.60	1.39	5.21
condenser duty (Megawatt)	–1.52	–65.73	–55.50	–13.79	–8.24	–8.63	–14.43
condenser temperature (°C)	9.80	128.82	127.15	–102.46	–149.45	–82.11	–44.94
distillate rate (kmol/h)	73.68	2523.71	1722.75	1233.23	173.57	1038.47	449.77
reboiler duty (Megawatt)	18.81	74.73	53.98	9.82	3.94	8.57	10.75
reboiler temperature (°C)	87.00	137.21	171.77	3.61	–79.51	–66.72	–4.06
bottom rate (kmol/h)	5660.77	3137.06	1414.31	4707.21	1059.66	21.19	137.70
tray spacing (m)	0.61	0.61	0.61	0.61	0.61	0.61	0.61
tray type	sieve	sieve	sieve	sieve	sieve	sieve	sieve
column height (m)	6.10	27.43	17.07	3.66	3.05	25.60	20.73
diameter (m)	2.79	4.44	3.64	3.77	2.43	2.52	3.76

**Figure 4:**
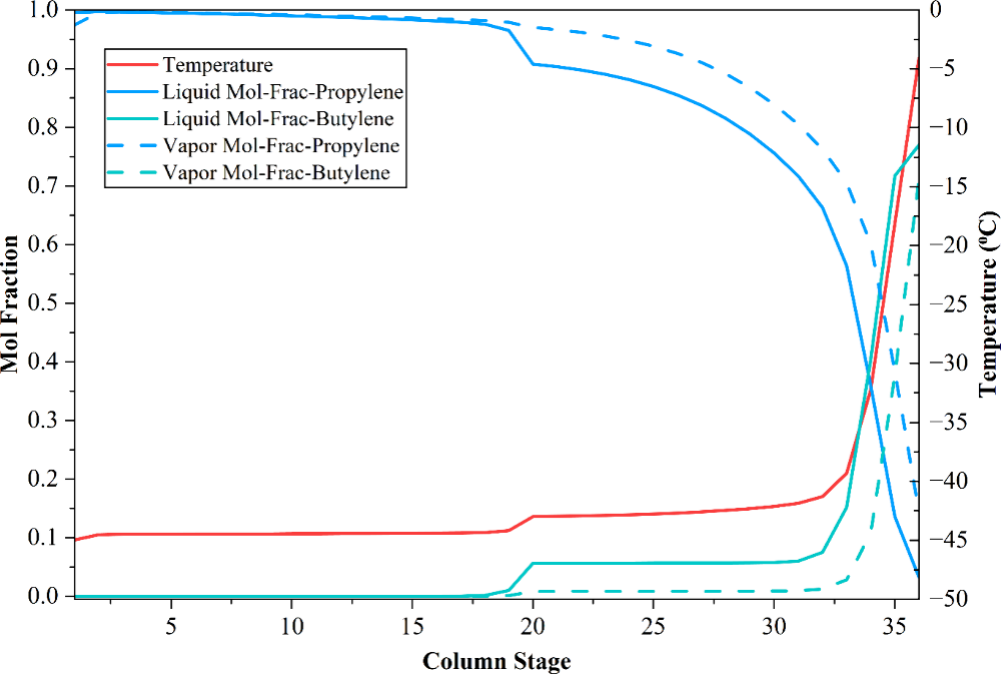
Temperature and composition profiles in the propylene separator column of the conventional process.

### 4.2 Novel Microwave Process

The novel microwave process employs only two distillation columns for product separation: the demethanizer (C-1) and the ethylene separator (C-2). The C-1 demethanizer column is designed with a feed stage at 11, a total of 21 stages, and a reflux ratio of 1.0. The column design parameters were optimized by adjusting the reflux ratio and the distillate-to-feed ratio to maximize the purity of methane in the overhead product stream. In contrast, the C-2 ethylene separator column is configured with a feed stage at 27, a total of 57 stages, and a reflux ratio of 5.0. Similar to the C-1 column, its design specifications were optimized by systematically varying the reflux and distillate-to-feed ratios to achieve a polymer-grade ethylene purity of 99.9% in the top product stream. The design and operational characteristics of these two columns differ from those of the demethanizer and ethylene purification columns used in the conventional process, primarily due to differences in feed composition and operating conditions. Figure 5 illustrates the vapor–liquid mole fraction profiles and temperature variations across the column stages for methane and ethylene in the demethanizer (C-1), while Figure 6 depicts corresponding profiles for ethylene and the bottom mixture in the ethylene separation column (C-2). The design specifications for both columns are summarized in Table 4. Both distillation columns utilize partial-vapor condensers, enhancing the energy efficiency within the separation process.

**Table 4:** Columns Specifications of the MW Novel Process

	C-1	C-2
purpose	distillation	distillation
no. of stages	21	57
feed stage	11	27
pressure (bar)	8	8
reflux ratio	0.24	2.60
condenser duty (Megawatt)	–15.27	–13.04
condenser temperature (°C)	–129.20	–58.40
distillate rate (kmol/h)	24,239.27	1571.25
reboiler duty (Megawatt)	1.48	1.43
reboiler temperature (°C)	–56.81	–20.18
bottom rate (kmol/h)	1689.37	118.12
tray spacing (m)	0.61	0.61
tray type	sieve	sieve
column height (m)	11.58	33.53
diameter (m)	6.28	3.27

**Figure 5:**
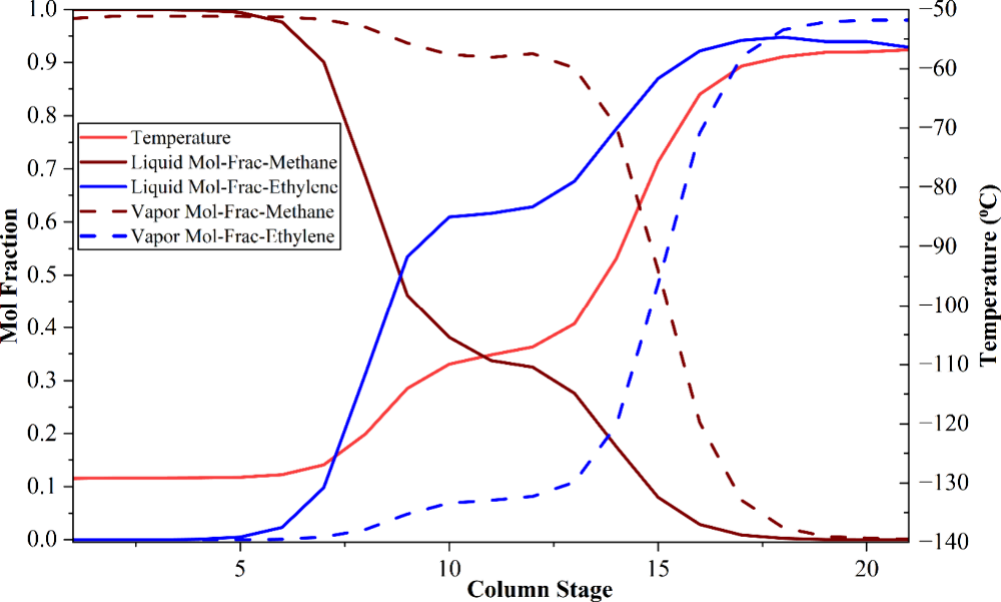
Temperature and composition profiles in the methane separator column of the MW novel process.

**Figure 6:**
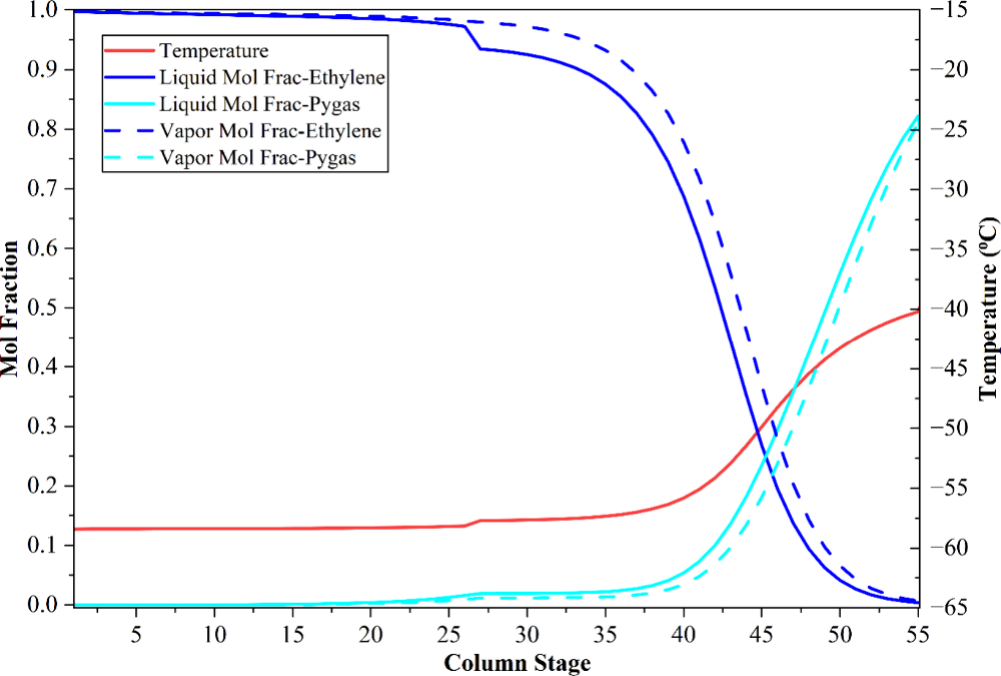
Temperature and composition profiles in the ethylene separator column of the MW novel process.

## 5 Results and Discussion

### 5.1 Technoeconomic Analysis of Ethylene Production

The APEA was utilized to perform the techno-economic evaluation of both the conventional and MW-assisted novel ethylene production processes. Two principal criteria were considered in the economic assessment: CAPEX and OPEX.^35^ CAPEX encompasses the total investment costs associated with equipment purchase, installation, piping, contingency allowances, and other relevant expenditures, whereas OPEX accounts for the costs of utilities, raw materials, and maintenance incurred during plant operation.^22^ The costs of major process equipment, including compressors, vessels, distillation columns, heaters, and heat exchangers, were estimated using APEA by mapping the corresponding equipment from the ASPEN Plus plant-wide simulation model.^35^ The utility cost data adopted in this study are summarized in Table 5, based on values reported in the literature.^1,22,35,36^ An electricity cost of USD 0.06 per kWh was applied in the analysis.^22^

**Table 5:** Cost of Utilities in the TEA Study

utility name	value	units
cooling water	0.378	USD/Gj
LP steam	4.55	USD/Gj
HP steam	5.66	USD/Gj
refrigeration1	4.77	USD/Gj
refrigeration2	8.49	USD/Gj
refrigeration3	14.12	USD/Gj
refrigeration4	15.95	USD/Gj
electricity	0.06	USD/KWh

The raw material costs and product prices used for the economic evaluation are listed in Table 6. Specifically, the methane price was obtained from Almaraz et al.,^22^ and the ethylene price was taken from Chen et al.^1^ It is important to note that the market prices of methane, ethylene, propylene, and pygas exhibit temporal and regional variability across the United States; hence, approximate average values were adopted for TEA in both cases.

**Table 6:** Cost of Raw Material and Products

classification	material name	price	units
raw material	methane	1.8	USD/MMBtu
products	ethylene	0.822	USD/kg
	propylene	0.95	USD/kg
	pygas	0.74	USD/kg

In addition, sensitivity analyses were conducted to evaluate the influence of key economic drivers on process feasibility, as discussed in subsequent sections. The investment parameters, including contingency, tax rate, internal rate of return (IRR), and annual operating hours, considered for both the conventional and MW novel processes are summarized in Table 7.^20,22,36^

**Table 7:** Key Economic Assumptions for TEA Study

investment parameters	value	unit
contingency	18	%
tax rate	40	%
desired internal rate of return	10	%
economic life of the project	20	years
salvage value	20	%
products, raw materials, and utility escalation	1	% per year
operating and labor maintenance escalation	1	% per year
operating hours	8000	hours
plant overhead	50	%
working capital percentage	12	%
startup period	20	weeks

#### 5.1.1 Technoeconomic Analysis of the Conventional Process

Table 8 illustrates the direct cost and CAPEX of the simulated conventional process. The cost of the reactors was about USD 51.94 MM, column costs were USD 27.4 MM, compressor costs were USD 37.24 MM, and heat exchangers cost about USD 92.94 MM.

**Table 8:** Total Direct Cost and CAPEX of the Conventional Process

major equipment cost	values (USD)
reactors	51,940,000
Columns	27,400,800
compressors	37,240,000
heat exchangers and heaters	92,938,932
contingencies	47,745,060
piping, installation, instrumentation and others	54,973,933
CAPEX	312,238,726

The TEA results for the conventional process are summarized in Table 9. The analysis indicated an estimated CAPEX of USD 312,238,726, while the total OPEX amounted to USD 202,945,248 per year. The OPEX was primarily composed of raw material costs of USD 46,253,754 per year and utility costs of USD 138,155,012 per year. Total product sales include USD 335,325,870 per year, which includes selling ethylene and pygas. The pygas can either be used as fuel or be separated into higher-value products such as benzene and toluene if there is a market for these products in the vicinity of the ethylene plant. However, separation of benzene and toluene from pygas requires additional investment in distillation systems, and so the capital and operating cost of these additional separations must be considered in the overall economics of the process. Overall, the net present value (NPV) of the conventional process was determined to be USD 265.73 million, and the corresponding levelized cost of ethylene (LCOE) was estimated to be USD 0.56 per kilogram.

**Table 9:** Summary of TEA of the Conventional Process

item	values (USD)
total capital investment (USD)	312,238,726
total operating cost (USD/yr)	202,945,248
cost of operating labor (USD/yr)	2,129,000
total product sales (USD/yr)	335,325,870
total raw material cost (USD/yr)	46,253,754
total utilities cost (USD/yr)	138,155,012
G and A Cost (USD/yr)	15,008,182
NPV (USD MM)	265.73
LCOE (USD/kg)	0.56

#### 5.1.2 Technoeconomic Analysis of the Novel Microwave Process

As the MW reactor technology represents a novel approach, the reactor cost estimation was derived from the combined costs of the magnetron system and the reactor vessel. The magnetron cost was evaluated based on the power requirement (kW) of the MW reactors, following the methodology outlined by Ogunniyan et al.^36^ The CAPEX breakdown for the simulated MW novel process is presented in Table 10.

**Table 10:** Total Direct Cost and CAPEX of the MW Novel Process

item	values (USD)
reactors	75,681,817
columns	26,657,900
compressors	110,880,000
heat exchangers and heaters	4,773,200
contingencies	49,006,024
piping, installation, instrumentation and others	53,486,122
CAPEX	320,485,063

The estimated reactor cost was approximately USD 75.68 million, which is higher than that of the conventional process due to the emerging nature of microwave-assisted reactor technology and its limited industrial deployment. The CAPEX of the MW reactors has been evaluated based on the price of a 200 kW microwave module, which is currently available commercially. Several modules of this size are utilized, and the total price of the microwave modular reactor system is estimated by this “numbering up” strategy. While this results in a microwave reactor price that is on the higher end, as we do not consider the economic benefits of making multiple units of the same size, this conservative strategy is feasible at the industrial scale. When microwave reactors are widely accepted in industry, increased competition among manufacturers would likely drive prices down and offer more cost-competitive options. Despite the relatively high initial CAPEX, the novel microwave process is projected to become cost-effective due to its lower OPEX, reduced utility demand, and enhanced energy efficiency. Additionally, the compressor cost was estimated at approximately USD 110.88 million, which is relatively high owing to the low single-pass methane conversion in the process. This necessitated a high recycle ratio for unconverted methane, thereby increasing the overall compressor capacity and cost requirements.

The TEA parameters for the novel microwave process are summarized in Table 11. The analysis indicated that the CAPEX of the plant was estimated at USD 320,485,063, while the total OPEX amounted to USD 153,501,346 per year. The OPEX comprised raw material costs of USD 46,253,754 per year and utility costs of USD 94,243,616 per year. Overall, the net present value (NPV) of the simulated MW novel process was calculated to be USD 505.81 million, and the corresponding LCOE was determined as USD 0.51 per kilogram. These results highlight the economic advantage and improved cost efficiency of the MW-based process relative to those of the conventional ethylene production system.

**Table 11:** Summary of TEA of the MW Novel Process

item	values (USD)
total capital investment (USD)	320,485,063
total operating cost (USD/yr)	153,501,346
cost of operating labor (USD/yr)	1,089,000
total product sales (USD/yr)	333,172,256
total raw material cost (USD/yr)	46,253,754
total utilities cost (USD/yr)	94,243,616
G and A Cost (USD/yr)	11,370,470
NPV (USD MM)	505.81
LCOE (USD/kg)	0.51

#### 5.1.3 Economic Comparison between the Conventional Process and the Novel Microwave Process

The TEA evaluated the financial performance of the processes over an economic lifetime of 20 years to assess their economic viability, estimating key indicators such as NPV and the LCOE. Figure 7 presents a comparative NPV analysis of the conventional process and the novel microwave process. The results revealed that the novel microwave process exhibited a significantly higher NPV compared with the conventional counterpart. Although the microwave system involved a higher initial capital investment due to the cost of the microwave reactor, its substantially lower OPEX and reduced utility consumption contributed to an improved overall economic performance. Consequently, the TEA results confirmed that the novel microwave process is economically competitive, with an LCOE of USD 0.51 per kilogram of ethylene, compared to USD 0.56 per kilogram for the conventional process.

**Figure 7:**
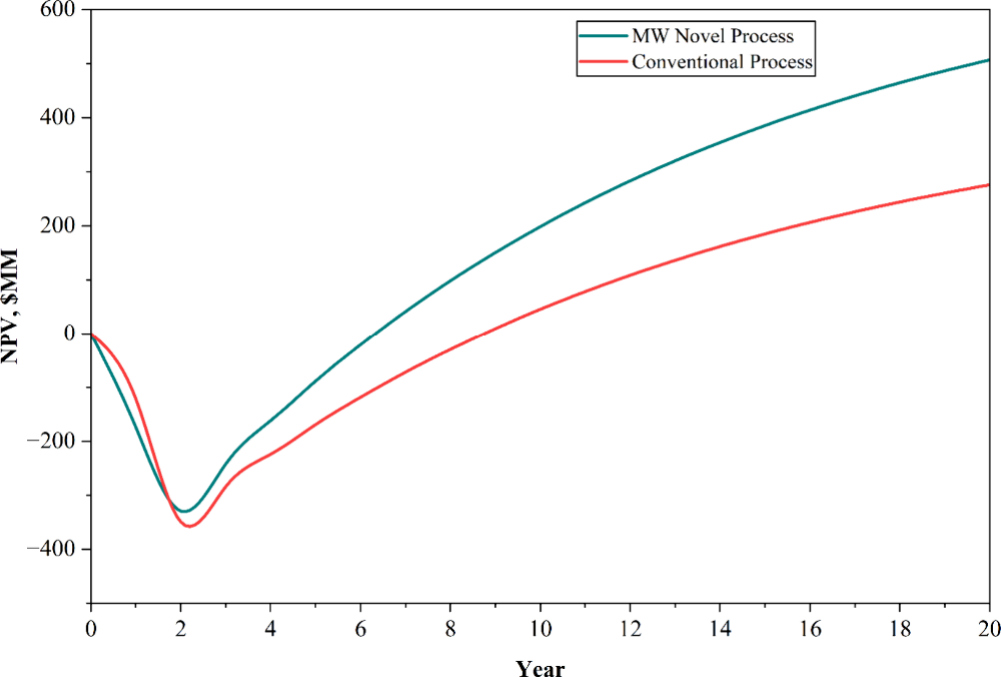
NPV comparative plot of conventional and novel processes.

The CAPEX breakdowns for both the conventional and novel microwave processes are presented in Figure 8. The comparison indicates that the novel MW process exhibited a higher reactor cost, as anticipated, primarily due to the high cost of the microwave generator (magnetrons). This elevated cost reflects the emerging nature of MW technology, which has yet to achieve large-scale industrial deployment and associated cost reductions. A significant difference was also observed in the compressor cost, which amounted to USD 110.88 million for the microwave process compared to USD 37.24 million for the conventional process. This disparity arises from the higher recycle ratio required in the microwave process to achieve greater overall methane-to-ethylene conversion efficiency. In contrast, the heater and heat exchanger costs were substantially lower in the microwave process (USD 4.77 million) compared to the conventional route (USD 92.94 million). This reduction is attributed to the elimination of high-temperature and high-pressure steam reforming operations in the MW process. The energy-efficient microwave system operated at comparatively lower temperatures and pressures, thereby reducing overall energy consumption and associated thermal equipment costs. Moreover, the piping, instrumentation, insulation, and ancillary system costs were higher for the conventional process, reflecting its greater process complexity. The novel microwave process, in contrast, featured a simpler process configuration with fewer unit operations. The contingency costs remained approximately equivalent for both cases, as the conventional ethylene production process is well established and the same 18% contingency factor is applied to both systems, except for the microwave reactor, which represents a novel technological component.

**Figure 8:**
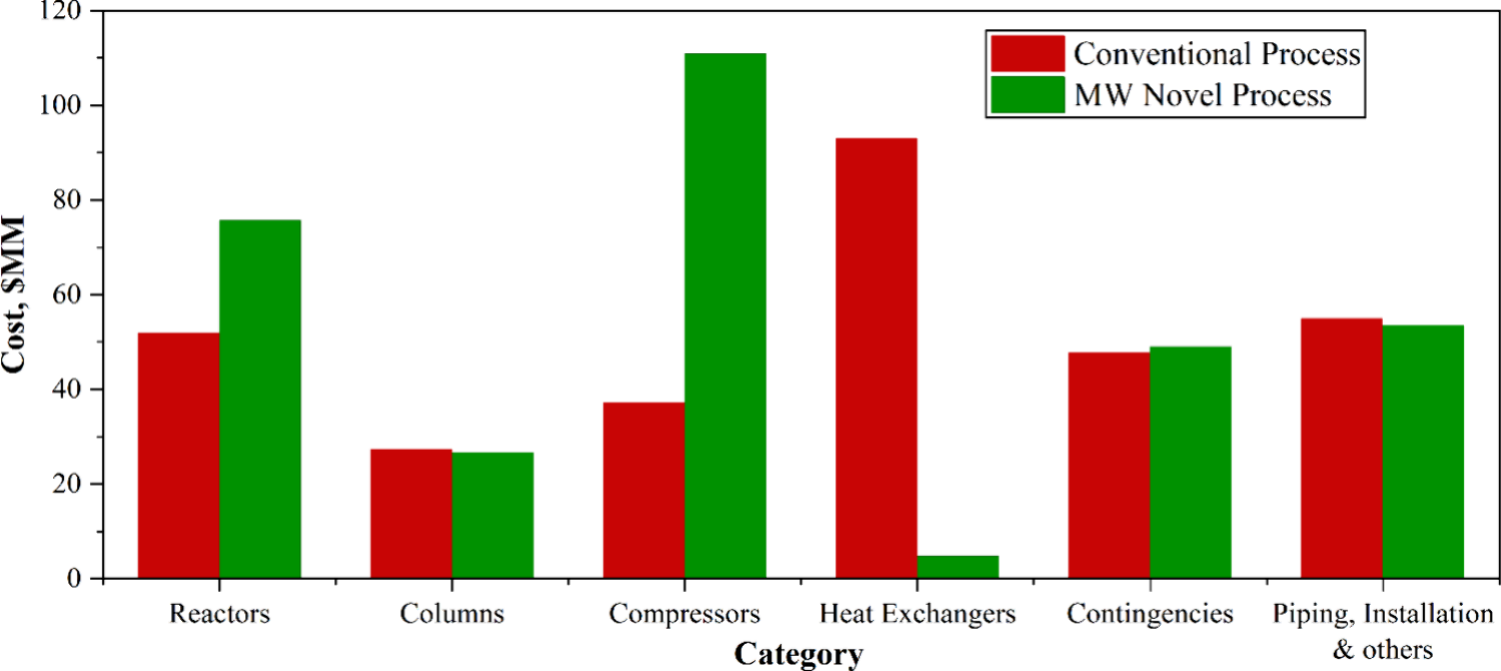
CAPEX breakdown for the conventional and novel processes.

Figure 9 presents the breakdown of operating costs across key categories, including raw materials, utilities, and general and administrative (G&A) expenses. As both the conventional and microwave processes utilize an identical methane feed rate of 67.781 t/h, the raw material costs remain equivalent in both cases. Notably, the utility cost for the novel microwave process was significantly lower than that of the conventional process. This reduction is primarily attributed to the electrification of the system, wherein approximately 88% of total utility consumption is in the form of electricity due to the use of electrified microwave reactors. The microwave process demonstrated a utility cost reduction of approximately USD 47.75 million per year, corresponding to a 34.56% decrease in overall utility consumption relative to the conventional configuration. Furthermore, the G&A expenses for the MW process were also lower than those of the conventional process, reflecting the simplified process design, fewer auxiliary systems, and reduced operational complexity associated with the MW-based ethylene production system.

**Figure 9:**
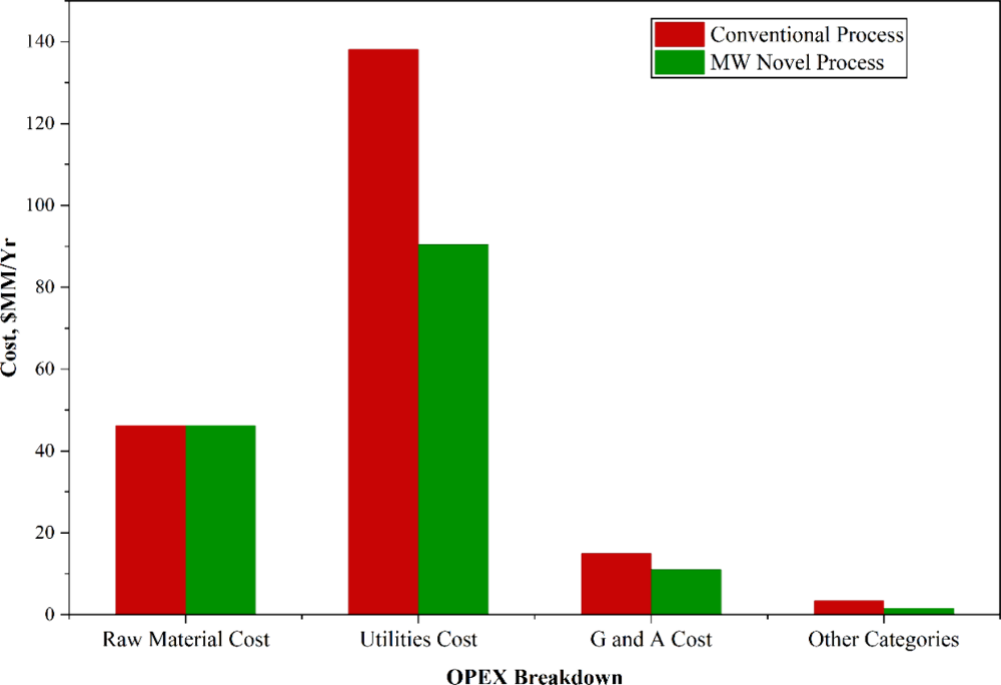
OPEX breakdown for conventional and novel processes.

To identify the dominant contributors to utility costs in both systems, a comparative analysis was performed, as illustrated in Figure 10. Figure 10a presents the utility cost distribution for the conventional process, while Figure 10b depicts the corresponding breakdown for the novel MW process. In the conventional process, electricity accounted for approximately 35.2% of total utility consumption, whereas in the microwave process, this share increased to 87.7%. This significant difference arose from the direct use of electricity as the primary energy source in the microwave process, where microwave reactors employ electromagnetic energy for the conversion of methane to ethylene. Specifically, about 56.6% of the total utility consumption in the MW process corresponded to electricity dedicated to microwave reactor operation, while an additional 31.1% was attributed to electricity use by compressors. This distribution underscores the high degree of process electrification and the energy transition potential of the novel MW system relative to the conventional configuration.

**Figure 10:**
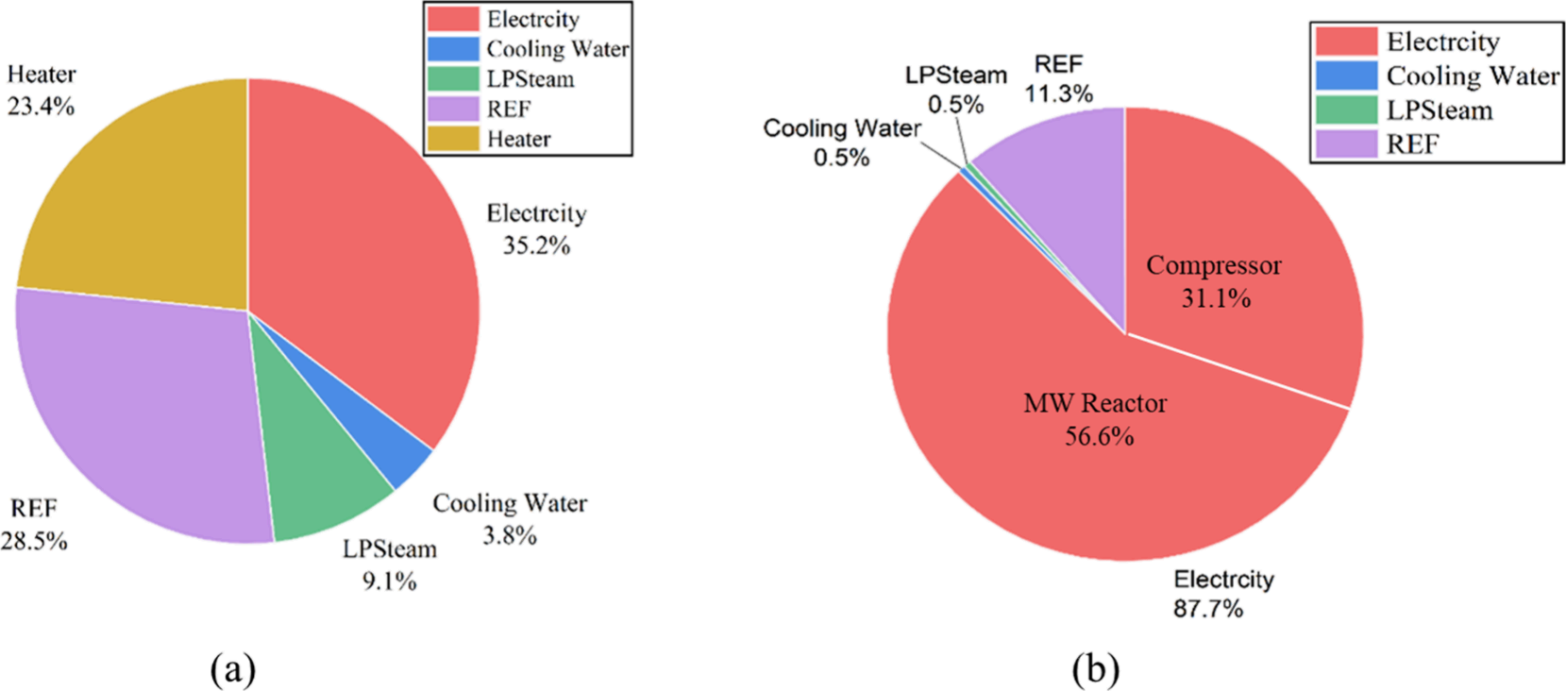
Utilities breakdown in (a) conventional process and (b) MW novel process.

#### 5.1.4 Sensitivity Analysis of the MW Novel Process

Based on CAPEX and OPEX analyses of the novel microwave process, it was identified that the reactor cost, electricity price, and raw material cost represent the primary economic drivers influencing overall process feasibility. Consequently, sensitivity analyses were performed with respect to these key parameters to account for cost uncertainties and to evaluate their respective impacts on the economic performance indicators, namely, the NPV and LCOE.

##### 5.1.4.1 Sensitivity Analysis on Reactor Cost

Sensitivity analysis on the microwave reactor cost is a critical component of this study, given that the technology is still in the developmental stage and the actual cost at a commercial scale remains uncertain. Since the microwave reactor cost directly contributes to the capital expenditure, an increase in its price can substantially affect the economic feasibility of the process. Specifically, a higher CAPEX reduces the initial cash flow, thereby decreasing the overall NPV of the project. To evaluate the influence of reactor cost on process economics, a parametric sensitivity analysis was conducted by varying the microwave reactor cost to 75, 50, and 25% of the base case reactor cost and by comparing it with the equivalent total reactor cost of the conventional base case. Table 12 presents the results of this sensitivity analysis, illustrating the impact of MW reactor cost variations on both NPV and LCOE. The results indicated that when the microwave reactor cost is reduced to 25% of the estimated cost (i.e., USD 18.92 million), the process achieved an NPV of USD 573.39 million and an LCOE of USD 0.47228/kg. When the MW reactor cost equaled that of the conventional process reactor (USD 51.94 million), the resulting NPV was USD 534.08 million, with a LCOE of USD 0.49626/kg. For comparison, the baseline case—with an MW reactor cost of USD 75.68 million yields an NPV of USD 505.81 million and an LCOE of USD 0.5135/kg, as illustrated in Figure 11. Additionally, the MW reactor cost of USD 280 MM results in an NPV for the MW-assisted process that is equivalent to that of the base-case process. These findings suggest that a reduction in MW reactor cost significantly enhances the economic attractiveness of the MW-based process, underscoring the importance of continued technological development and cost optimization for industrial implementation.

**Table 12:** Results of Sensitivity Analysis on the MW Reactor Cost

case	MW cost (USD MM)	NPV (USD MM)	LCOE (USD/kg)
MWR cost 25%	18.92	573.39	0.472
MWR cost 50%	37.84	550.86	0.486
SMR reactor price	51.94	534.08	0.496
MWR cost 75%	56.76	528.33	0.499
MWR cost 100%	75.68	505.81	0.513

**Figure 11:**
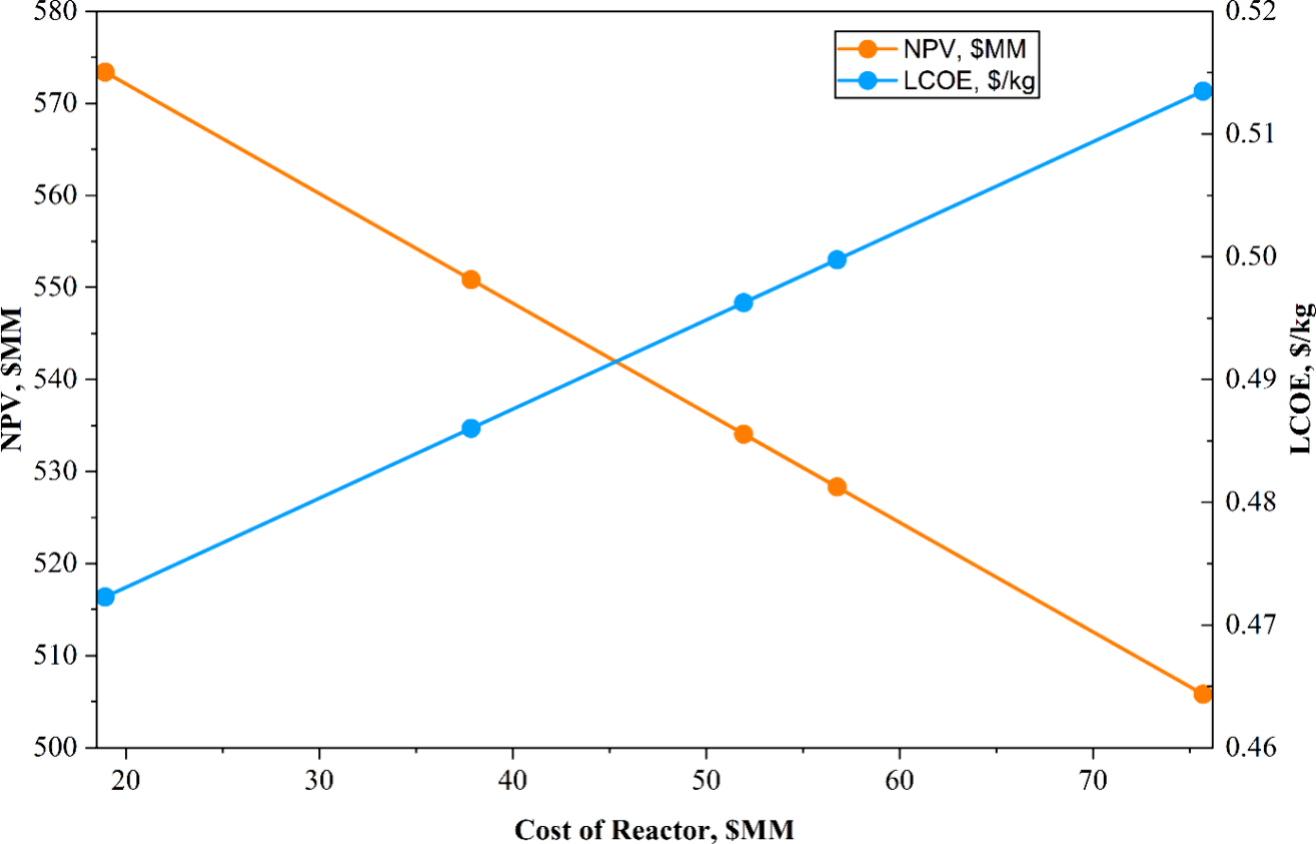
Sensitivity analysis of the cost of the reactor.

##### 5.1.4.2 Sensitivity Analysis on Raw Material Cost

Since methane serves as the sole feedstock for the novel MW process, its price exerts a dominant influence on the OPEX. Any increase in methane price directly and significantly elevates the LCOE, which consequently leads to a sharp decline in the NPV, thereby adversely affecting the overall economic feasibility of the process. To quantify this effect, a sensitivity analysis was performed by varying the methane price between USD 1.8 and USD 4.4 per MMBtu, and the corresponding impacts on NPV and LCOE were calculated, as summarized in Table 13. The analysis revealed that at a methane price of USD 1.8/MMBtu, the process yielded the highest NPV of USD 505.81 million and the lowest LCOE of USD 0.51/kg, representing the most favorable economic scenario. Conversely, when the methane price increased to USD 4.4/MMBtu, the NPV decreased to USD 146.93 million, and the LCOE rose to USD 0.732/kg, indicating the least favorable case, as depicted in Figure 12. The trend illustrated in Figure 12 clearly demonstrates that further increases in methane price would render the project economically less attractive, potentially resulting in a negative NPV. Additionally, the natural gas feed price of USD 3.55/MMBtu results in an NPV for the MW-assisted process that is equivalent to that of the base-case process. This outcome highlights the strong dependency of the microwave process economics on methane price fluctuations and underscores the importance of stable and low-cost feedstock supply to ensure its competitiveness relative to the conventional ethylene production pathway.

**Table 13:** Results of Sensitivity Analysis on the Raw Material Cost of the MW Novel Process

methane price (USD/MMBtu)	NPV (USD MM)	LCOE (USD/kg)
1.8	505.81	0.513
2.4	423.08	0.563
2.8	367.73	0.597
3.2	312.38	0.631
3.6	257.33	0.665
4	201.98	0.698
4.4	146.93	0.732

**Figure 12:**
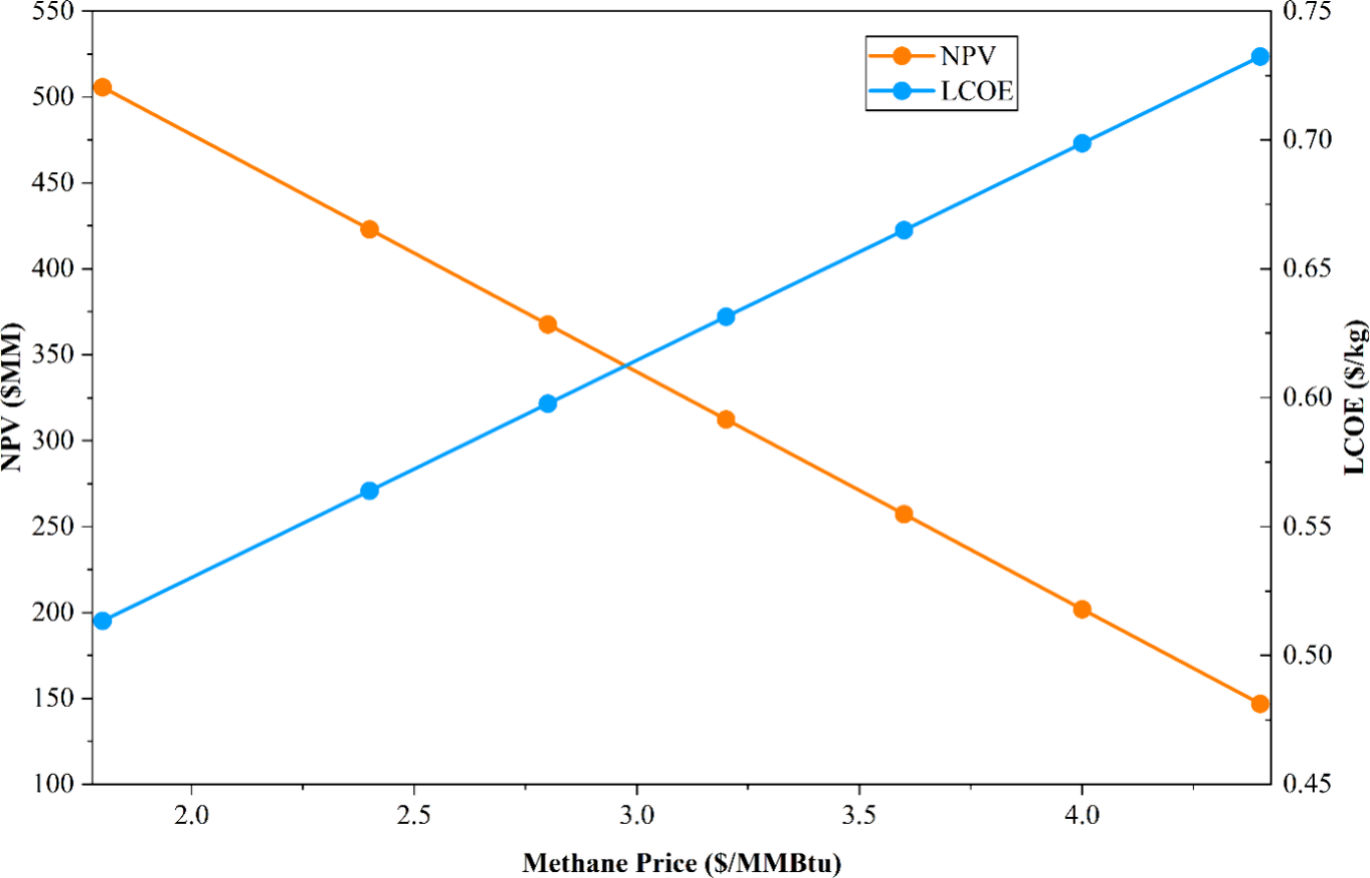
Sensitivity analysis of the raw material cost.

##### 5.1.4.3 Sensitivity Analysis on Electricity Cost

The sensitivity analysis on electricity price is of particular significance in this study, as it reflects the broader industrial transition toward electrification. In the novel microwave process, electricity constituted approximately 87.7% of the total utility consumption, making it a critical economic driver that substantially influences both the NPV and the LCOE. Consequently, any increase in electricity price directly elevates the OPEX and reduces the profit margin per unit of ethylene produced. To evaluate this dependency, a sensitivity analysis was conducted by varying the electricity price from USD 0.02/kWh to USD 0.12/kWh, and the resulting impact on NPV and LCOE was estimated, as summarized in Table 14 and Figure 13. The analysis reveals that as electricity prices increase, the NPV decreases correspondingly, while the LCOE rises proportionally. Specifically, at an electricity price of USD 0.02/kWh, the process achieved an NPV exceeding USD 803.5 million and a low LCOE of USD 0.331/kg, representing a highly favorable economic scenario. Conversely, when the electricity price reached USD 0.12/kWh, the NPV declined sharply to USD 59.26 million or even negative values, accompanied by a high LCOE of USD 0.785/kg, rendering the process economically unfeasible. Additionally, an electricity price of USD 0.092/kWh results in an NPV for the MW-assisted process that is equivalent to that of the base-case process. These findings emphasize that the economic competitiveness of the MW-assisted process is highly sensitive to the electricity pricing. Therefore, the availability of low-cost renewable electricity is essential to ensuring the long-term economic viability and sustainability of this electrified chemical production pathway.

**Table 14:** Results of Sensitivity Analysis on the Electricity Cost of the MW Novel Process

electricity price (USD/kWh)	NPV (USD MM)	LCOE (USD/kg)
0.02	803.50	0.331
0.04	654.66	0.422
0.06	505.81	0.513
0.08	356.96	0.604
0.1	208.11	0.695
0.12	59.26	0.785

**Figure 13:**
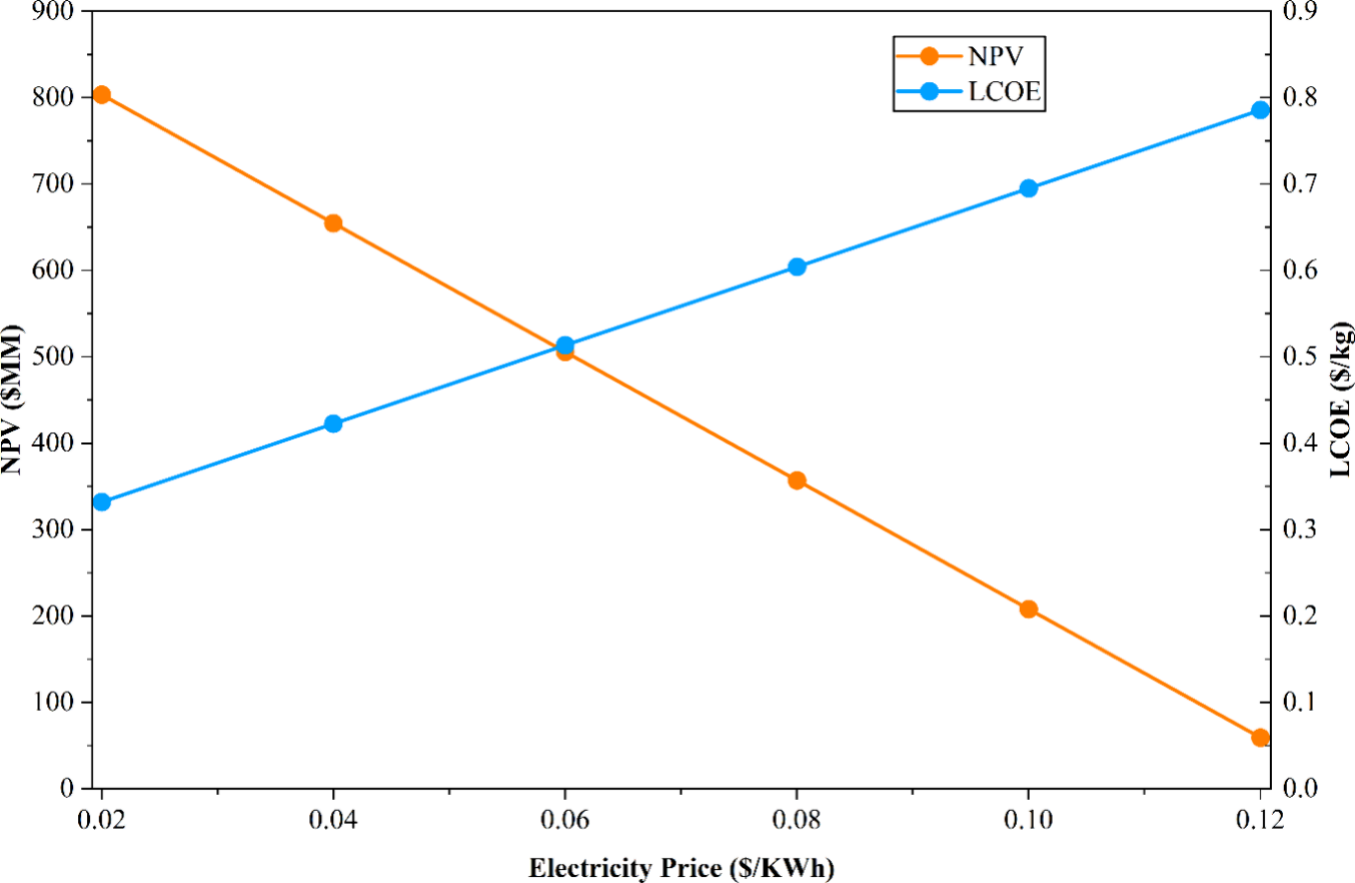
Sensitivity analysis of the electricity cost.

##### 5.1.4.4 Sensitivity Analysis on Ethylene Selling Price

The selling price of ethylene, the primary product, represents the most sensitive factor influencing the project financing and economic viability. Variations in the selling price directly affect the net cash flow, thereby strongly impacting the NPV of the project. For the economic analysis, a baseline ethylene price of USD 0.822/kg was assumed. To capture the potential market fluctuations, a sensitivity analysis was performed over a price range of USD 0.5 to USD 1.1/kg. As summarized in Table 15, a lower ethylene price of USD 0.5/kg resulted in a negative NPV of – USD 22.14 million, whereas USD 0.6/kg represented the minimum selling price required to achieve a positive NPV of USD 141.14 million. Conversely, at a higher selling price of USD 1.1/kg, the NPV reached a maximum value of USD 961.61 million, indicating significantly enhanced project profitability. Figure 14 illustrates the corresponding trend of NPV variation with ethylene selling price, highlighting the strong sensitivity of the project’s economic performance to product pricing.

**Table 15:** Results of Sensitivity Analysis on Ethylene Selling Price

ethylene selling price (USD/kg)	NPV (USD MM)
0.500	–22.14
0.600	141.82
0.700	305.78
0.822	505.81
0.900	633.69
1.000	797.65
1.100	961.61

**Figure 14:**
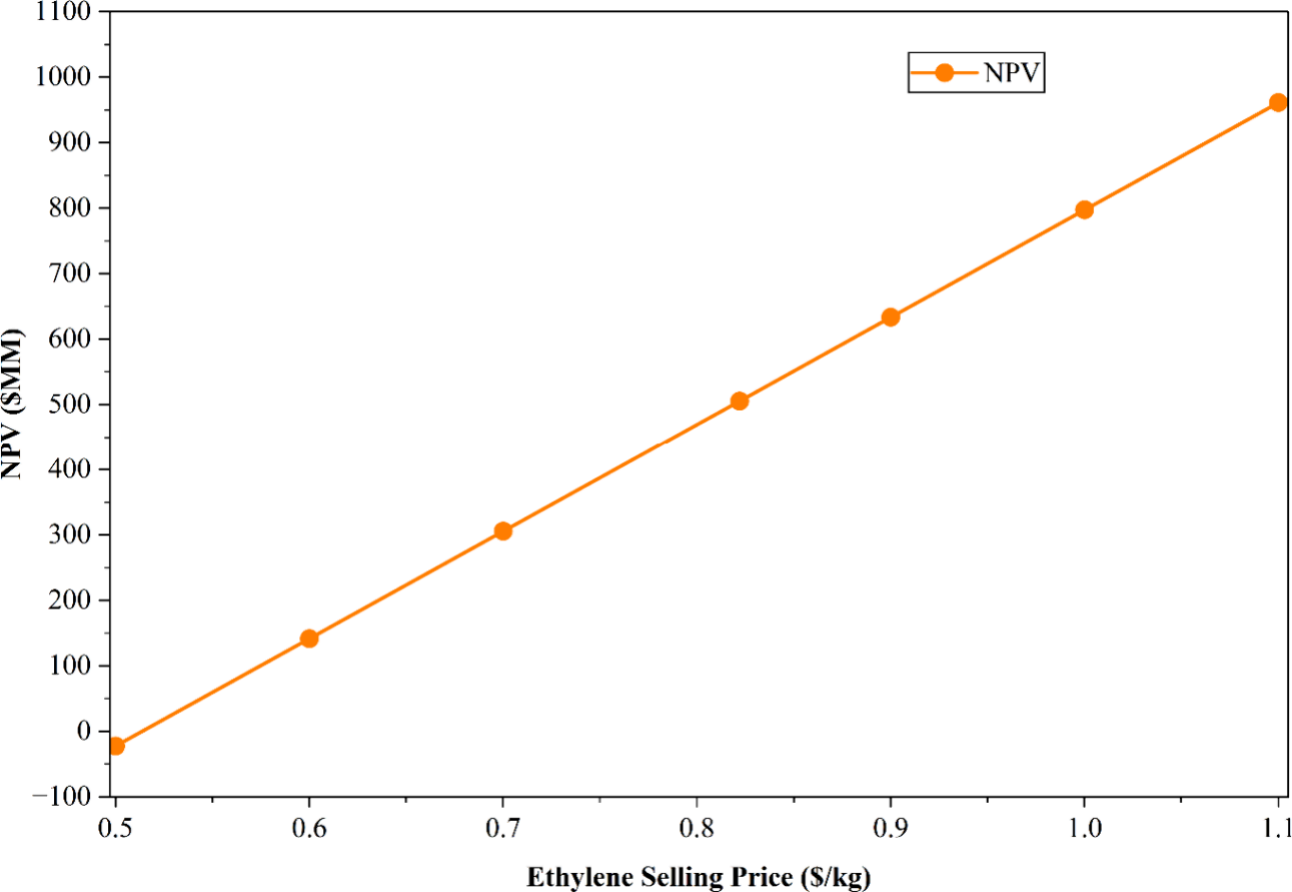
Sensitivity analysis on ethylene selling price.

##### 5.1.4.5 Summary of Sensitivity Analysis

The LCOE difference (USD 0.51/kg vs USD 0.56/kg) results in an NPV difference of USD 240 MM between the base case and the MW-assisted process. Sensitivity analysis indicates that the economic outcome strongly depends on dominant cost drivers, including OPEX factors such as raw material and electricity prices, as well as CAPEX factors such as MW-reactor cost. For instance, the NPV of the MW-assisted becomes equal to the base case if all of the following happen: (1) the electricity price increases from USD 0.06/kWh to USD 0.092/kWh, (2) MW-reactor costs increase from USD 75.68 to USD 280 MM, and (3) natural gas feed prices increase from USD 1.88/MMBtu up to USD 3.55/MMBtu. These ranges demonstrate substantial potential for the microwave process to be profitable.

The sensitivity analysis can also be used to determine the cost of different factors that result in a break-even economic scenario (i.e., selling price equal to the cost of manufacture), if only one factor is changed while the others are kept at their base values. It is seen that zero profitability of the novel microwave process happens if the electricity price is USD 0.128/kWh, the MW-reactor cost is USD 500.5 MM, or the raw material prices are up to USD 5.46/MMBtu. When individual factors move beyond these extreme bounds, the MW-assisted process becomes economically nonviable. Current market prices indicate that the proposed system remains resilient under realistic market fluctuations.

### 5.2 Modular Plant Simulations of the Novel Microwave Process

Modular reactors are attracting increasing interest due to their potential to enhance the grid stability of renewable power generation.^37^ Process intensification, achieved through innovative, turn-down–friendly reactor designs, facilitates modular deployment, thereby increasing operational flexibility in response to fluctuations in demand and supply, which can yield significant economic benefits.^38^ Modular plants have been proposed for methanol production from shale gas in remote locations, considering both economic and environmental advantages.^39^ In this context, the present study extended the investigation to assess the economic feasibility of the novel microwave process under turn-down capacity conditions, reflecting the potential for modular plant operation. The nominal methane feed rate of 67.781 t/h was systematically reduced to turn-down capacities of 50, 25, 10, and 5 t/h, respectively. For each capacity scenario, the reactor cost of the MW novel process was estimated, and the TEA was conducted following the same methodology as that for the full-scale process. The results, summarized in Table 16, indicate that even at a methane feed rate of 5 t/h, the process achieves a positive NPV of USD 28.44 million, producing 3.257 t/h of ethylene, thereby demonstrating the financial viability of modular deployment. These findings validate the potential of novel microwave modular plants for ethylene production from methane, particularly in locations with limited feedstock availability. Figure 15 illustrates the relationship between NPV, ethylene production, and modular plant feed capacity. While ethylene production scales proportionally with plant capacity, the NPV exhibits a nonlinear relationship, reflecting the complex interplay between economies of scale and capital and operational costs in modular configurations.

**Table 16:** Modular Plant TEA Results

feed capacity (t/h)	MW cost (USD MM)	ethylene production (t/h)	NPV (USD MM)
5	4.97	3.257	28.44
10	10.04	6.51	76.80
25	24.50	16.277	251.18
50	49.96	32.54	448.71
67.78	75.68	44.076	505.81

**Figure 15:**
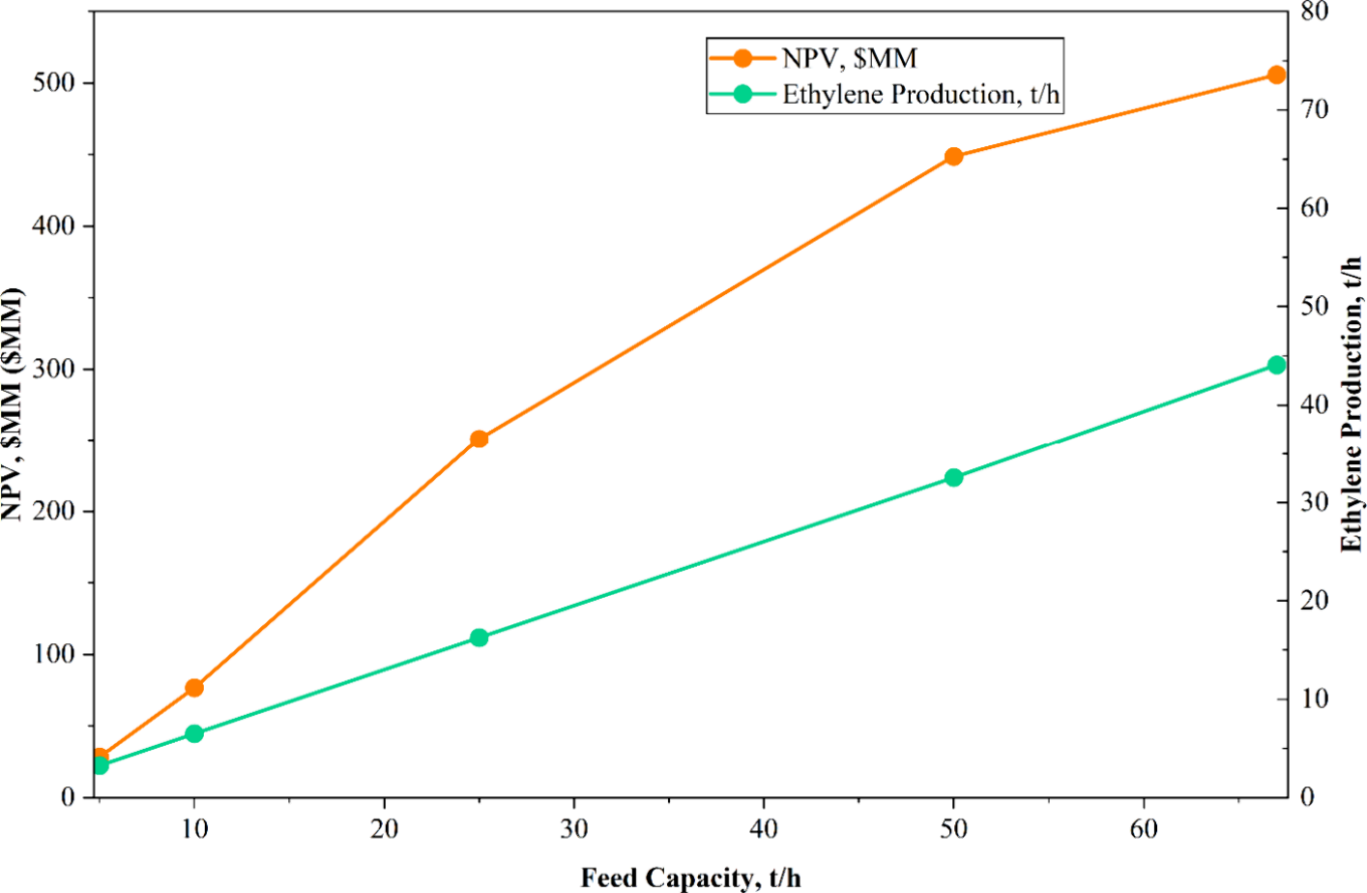
NPV and ethylene production vs modular plant capacity of MW novel process.

A 5 t/h feed capacity modular MW plant requires 12,500 kW of electricity, which could be supplied by a solar power plant, indicating the potential for coupling modular MW reactors with renewable microgrids.^40^ Such integrations could enhance sustainability and operational flexibility.

### 5.3 Energy Efficiency and Impact of Electricity Source on Carbon Footprint

MW-enhanced catalytic conversion of methane to ethylene, with a reduction in hot-utility demand by over 50% and cold-utility requirements by up to 80% compared with those of the conventional process, demonstrates that the MW reactor technology is highly energy-efficient. In parallel, a preliminary gate-to-gate carbon footprint analysis has shown a 40% reduction in CO_2_ emissions for the MW process compared to the conventional base-case process. This demonstrates that the MW process has a significantly lower carbon footprint compared to the conventional route, highlighting its potential environmental advantage.

Since the MW-assisted process relies heavily on electricity (87.7% of total utilities), the carbon footprint is strongly influenced by the source of electricity. According to Varun et al.,^41^ electricity from fossil-fuel power plants has a significantly higher carbon footprint, with natural gas–powered plants at 607.6 g CO_2_ eq/kWh and coal-fired plants at 75.3 g CO_2_ eq/kWh. In contrast, renewable sources such as wind and solar have much lower footprints, ranging from 9.7 to 123.7 g CO_2_ eq/kWh for wind and 53.4–50 g CO_2_ eq/kWh for solar.^41^ Therefore, if a portion of the electricity used in the MW process comes from fossil sources, then the resulting indirect CO_2_ emissions could be significant, potentially reducing the environmental benefits of the MW-assisted route. Since electricity accounts for most of the utility consumption, shifting from fossil-based grid power to renewable sources could substantially lower the carbon footprint. In fact, if the MW process were powered entirely by renewables, then CO_2_ emissions associated with electricity use could potentially be reduced by over 80%. On the other hand, if a dedicated natural-gas-fired plant is utilized for a 5 t/h feed in an MW plant that requires 12,500 kW of electricity, this plant would generate 7.595 t/h of CO_2_, which provides the potential to utilize carbon capture and utilization (CCU) systems to enhance sustainability and process flexibility.

The MW-assisted process reduces direct CO_2_ emissions compared with conventional fired-heater ethylene production, but this advantage comes with potential environmental trade-offs. Its high electricity demand can lead to significant indirect emissions if fossil-fuel-based power is used. In contrast, cooling requirements represent only 11.3% of total utilities in the MW process compared with 28.4% in the conventional process. Thus, while combustion-related point-source emissions are reduced, some emissions are effectively shifted upstream of electricity generation.

Overall, MW-assisted ethylene production can reduce CO_2_ emissions by over 80% and, in terms of energy efficiency, lower hot-utility demand by more than 50% and cut cold-utility requirements by up to 80% compared with the conventional process, collectively supporting industrial decarbonization and advancing the UN 2030 Agenda (Goal 7) for sustainable development.^42^ Furthermore, the MW-assisted ethylene production supports Goal 12 ^42^ by using natural gas more efficiently, reducing emissions, and replacing conventional fossil-fuel-based heating through industrial electrification, thereby minimizing adverse impacts and promoting sustainable energy consumption through the development of novel, cleaner chemical production patterns.

## 6 Conclusions

This study demonstrates the economic feasibility of a novel microwave process for ethylene production from methane at an industrial scale, relative to conventional processes. The economic evaluation indicated that the novel microwave process achieved an NPV of USD 505.71 million, substantially higher than the USD 265.73 million NPV of the conventional process. The techno-economic analysis further confirmed the economic competitiveness of the novel microwave process, with a levelized cost of ethylene of USD 0.5135/kg, compared to USD 0.5648/kg for the conventional base case. Despite the promising economics, the high initial cost of the microwave reactor remains a key challenge; however, this cost is expected to decrease as the technology becomes more widely adopted within the context of industrial electrification. Notably, the process is predominantly electricity-driven, with 87.7% of total utility consumption attributed to electricity, in contrast to the conventional process, highlighting its potential to support the transition toward electrified chemical manufacturing. It should be noted that scaling MW-assisted reactors to an industrial scale requires updated policies, including guidelines for MW frequency exposure. Operating MW reactors at higher pressure could reduce the compressor pressure ratio, lowering energy consumption, and this will be explored in future studies.
